# Infection of Wounds by Perspiration of the Hands

**Published:** 1901-05

**Authors:** 


### Abstracts and Selections.

### Infection of Wounds by Perspiration of the Hands.

M. E. Genevet has adopted this method of sterilizing his hands for surgical procedures: Washing and scrubbing with soap and water for fifteen minutes, then washing with a pad of sterilized gauze impregnated with ether, then rinsing them with ninety-five per cent. alcohol, and finally washing them with sterilized water for five minutes. No cultures could be obtained from the hands after this method of disinfection. But when the hands were made to perspire freely after such washing, the transparent fluid always yielded a pure and virulent culture of the *Staphylococcus albus*. The author concludes: (1) that it is possible to obtain absolute sterilization of the hands; (2) that as soon as perspiration of the hands begins, infection of the wound is possible; (3) that, as it is impossible to disinfect the operative area, the lips of the cutaneous wound should always be protected; (4) disinfection of the hands should be completed by their immersion for ten minutes in a two per cent. solution of tannin to inhibit the sweating; (5) gloves should be worn in septic operations where mere manual dexterity is not an important factor—[*Gazette hebdomadaire de médecine et de chirurgie.*]—*N. Y. Medical Journal.*

